# MicroRNAs as diagnostic biomarkers for Tuberculosis: A systematic review and meta- analysis

**DOI:** 10.3389/fimmu.2022.954396

**Published:** 2022-09-27

**Authors:** Evangeline Ann Daniel, Balakumaran Sathiyamani, Kannan Thiruvengadam, Sandhya Vivekanandan, Hemanathan Vembuli, Luke Elizabeth Hanna

**Affiliations:** ^1^ Department of Virology and Biotechnology, ICMR- National Institute for Research in Tuberculosis, Chennai, India; ^2^ University of Madras, Chennai, India; ^3^ Department of Statistics, Epidemiology Unit, ICMR-National Institute for Research in Tuberculosis, Chennai, India

**Keywords:** tuberculosis, miRNA, diagnosis, biomarker, immune modulation, *mycobacterium tuberculosis*

## Abstract

**Background:**

The early diagnosis of tuberculosis using novel non-sputum-based biomarkers is of high priority in the End TB strategy. MicroRNAs (miRNAs) are significant regulators of TB pathogenesis and their differential expression pattern among healthy, latent, and active TB population has revealed their potentiality as biomarkers in recent studies. Thus, we systematically reviewed and performed a meta-analysis on the role of host miRNAs in TB diagnosis. We also reviewed the involvement of miRNAs in the immune response to *Mycobacterium tuberculosis* (*Mtb*).

**Methods:**

Pubmed, Ovid and Cochrane databases were searched to retrieve published literature from 2000 to 2020 using predefined keywords. We screened relevant studies based on inclusion and exclusion criteria and the included studies were assessed for their quality using STARD guidelines and QUADAS-2 tool. Funnel plots were constructed to assess the publication bias. The heterogeneity of studies and overall pooled results of sensitivity, specificity and DOR were determined using forest plots.

**Results:**

We retrieved a total of 447 studies collectively from all the databases, out of which 21 studies were included for qualitative analysis. In these studies, miR-29, miR-31, miR-125b, miR146a and miR-155 were consistently reported. The overall sensitivity, specificity and DOR of these miRNAs were found to be 87.9% (81.7-92.2), 81.2% (74.5-86.5) and 43.1(20.3-91.3) respectively. Among these, miR-31 had the maximum diagnostic accuracy, with a sensitivity of 96% (89.7-98.5), specificity of 89% (81.2-93.8) and DOR of 345.9 (90.2-1326.3), meeting the minimal target product profile (TPP) for TB diagnostics.

**Conclusion:**

miRNAs can thus be exploited as potential biomarkers for rapid detection of tuberculosis as evident from their diagnostic performance.

**Systematic Review Registration:**

https://www.crd.york.ac.uk/prospero/display_record.php?ID=CRD42021226559 PROSPERO (CRD42021226559).

## Introduction

Tuberculosis (TB) remains as one of the major causes of global morbidity and mortality. Among one-fourth of the world's population latently (asymptomatically) infected with *Mycobacterium tuberculosis (Mtb)*, 5 to 10% bear the risk of developing active TB during their lifetime (1, 2). Identifying and treating this pool of individuals could contribute significantly to TB elimination. An ideal TB biomarker must exhibit explicit identification of subjects with *Mtb* infection, differentiation between active TB disease and Latent Tuberculosis infection (LTBI), and possibly predict for future progression to active disease (3). Research in the past few years has focused on the identification of a broad range of biomarkers and biosignatures using transcriptomic, metabolomic, and proteomic approaches in various sample types, such as blood, sputum, and urine. Maclean et al. recently published a systematic review encompassing various biomarkers for detecting active TB (4). Independent systematic reviews on the diagnostic accuracy of transcriptomic signatures derived from the whole blood of host for diagnosis of active TB (5, 6) and incipient TB (7), and the utility of biomarkers like IFN-γ (8–10), IP-10 (11, 12), LAM (13, 14), and IL-2 (15) for the detection of TB infection and disease are also available. More recently, systematic reviews on *Mtb*- specific cytokine biomarkers that can differentiate between active TB and LTBI have been published (16, 17). Reviews on the diagnostic potential of novel *Mtb* antigens (18) and other informative reviews on current TB biomarker research are also available (19, 20). However, none of these reviews provide accurate data to guide the development of a point-of-care test.

MicroRNAs (miRNAs) are highly conserved, small, non-coding RNAs of ~18-25 nucleotides that are involved in regulating gene expression at the post-transcriptional level (21). The seed region of miRNAs typically spanning six nucleotides (residues 2-8 at the 5’ end) binds to the target mRNA in the 3’ untranslated region (UTR) resulting in translational repression or mRNA degradation (22). In this way, miRNAs modulate several key functions including cell growth, differentiation, and apoptosis. Most importantly, miRNAs regulate the production of a myriad of cytokines and chemokines that modulate the host immune response during various disease states, and aberrant miRNA expression patterns have been associated with the pathogenesis of many diseases (23). Since its binding does not require perfect complementarity, a single mRNA can be acted upon by multiple miRNAs and a single miRNA can target hundreds of mRNAs resulting in enhanced gene regulation (24). Till date, around 2600 functional miRNAs targeting almost 60% of human genes have been identified (25). Beyond their presence in the cellular milieu, miRNAs are also released into circulation and can be identified in different body fluids. Most of the released miRNAs are complexed to proteins like Argonaute2 (AGO2), and are therefore remarkably stable in circulation and resistant to RNases, extreme temperatures, acidic, and alkaline conditions, as well as repeated freeze-thaw cycles (26). With their high stability, ease of recovery, substantial levels of sensitivity and specificity and translational potential as a point of care test, miRNAs are considered to be promising diagnostic biomarkers.

**Figure 1:**
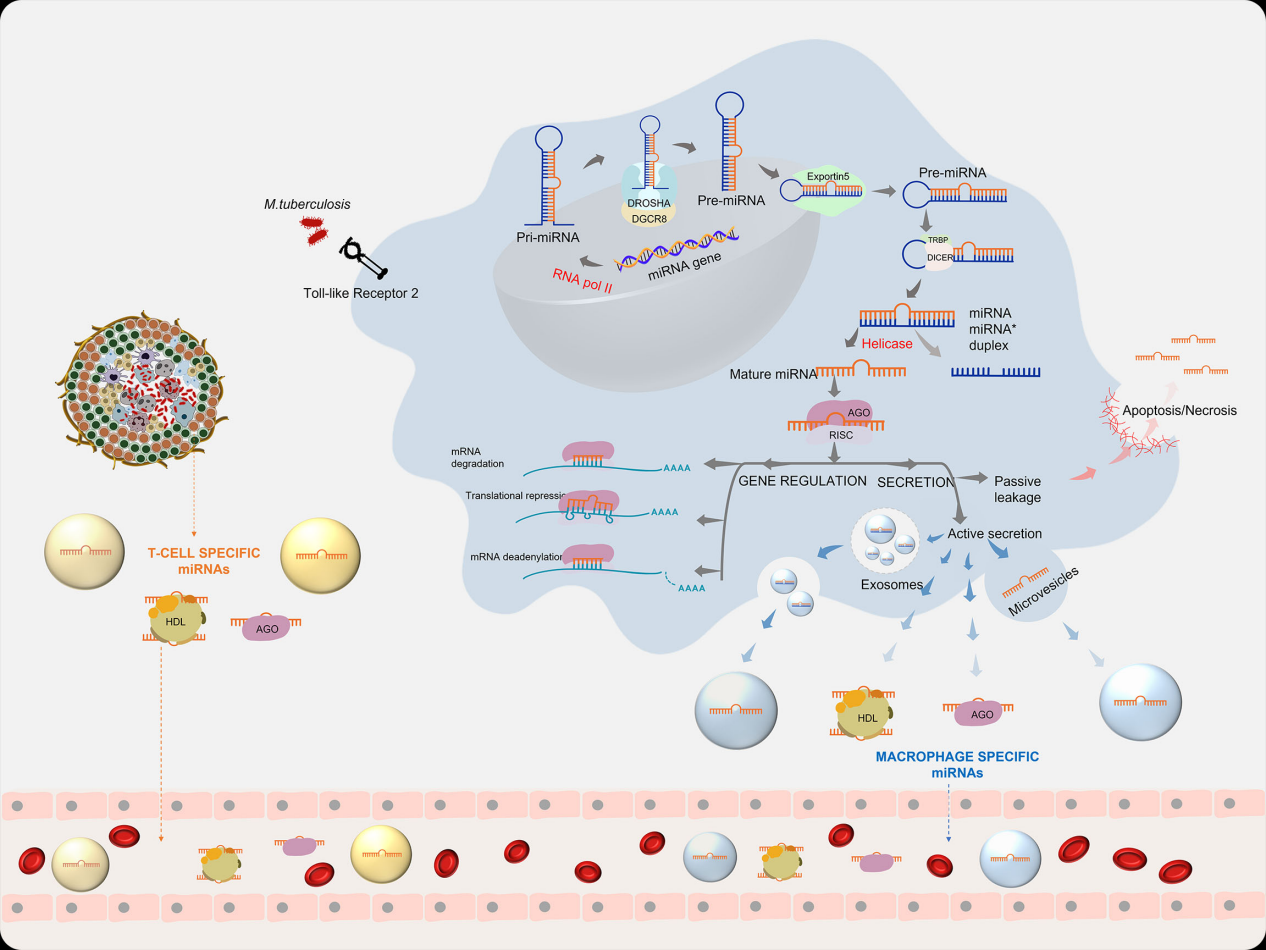
miRNA biogenesis during *Mycobacterium tuberculosis* infection: *Mtb* infection leads to the initiation of miRNA production, which after processing gives rise to mature miRNAs. Mature miRNAs can regulate gene expression and can be secreted into the extra cellular environment (30).

Upon infection with *Mtb*, the host cells repattern their immune response for the defence arsenal. *Mtb* on the other hand tries to subvert the host defenses for its intracellular survival by manipulating the host miRNA profile (27). This reprogramming involves the regulation of various biological processes by miRNAs. The first line of host defence to *Mtb* infection begins when the Pattern Recognition Receptors (PRRs), majorly the Toll-like receptors (TLRs), present on the surface of macrophages and dendritic cells recognise the pathogen-associated molecular patterns (PAMPs). Stimulation of TLRs leads to the induction of miRNA biogenesis. The miRNA coding transcripts are transcribed as primary- miRNA (pri-miRNA >100 nt) by RNA polymerase II. Pri-miRNA is cleaved by a microprocessor complex comprising of an RNase III endonuclease (drosha) and its essential cofactor DGCR8 (Pasha) into precursor miRNA (pre-miRNA) (60-70 nt). The pre-miRNA is loaded onto Exportin 5 and transported to the cytoplasm where it is further processed by Dicer, a ribonuclease III enzyme, with the help of its cofactor TRBP into a duplex of 20-25 nt. A helicase enzyme unwinds the duplex into a mature miRNA and a lesser expressed passenger strand (miRNA*). Under some conditions, mature miRNA can also arise from miRNA*. The mature miRNA is now integrated into a multiprotein complex called RNA-induced silencing complex (RISC) and is equipped for gene regulation (28). Gene regulation by miRNA takes place by three mechanisms, based on the complementarity of the miRNA with its target mRNA. A perfect binding results in mRNA degradation, while a near perfect/partial complementarity leads to translational repression. mRNA deadenylation leading to destability is a third mechanism which accelerates mRNA degradation (29). Through either of these ways, gene regulation by miRNA inside the macrophage leads to the production of multiple cytokines and chemokines that trigger the host innate immune response. TB infection-specific miRNA also arise from other immune cells contained within the granuloma and prime the adaptive immune response. Besides directly controlling gene expression, these miRNA can also be secreted into the extracellular milieu either by passive leakage through cell apoptosis/necrosis or by active secretion *via* encapsulation within microvesicles/exosomes and by binding to high-density lipoproteins (HDL) or AGO2 proteins. (30) This process can give rise to highly stable *Mtb*-associated miRNA signatures in various biological fluids (**Figure 1**).

**Figure 2:**
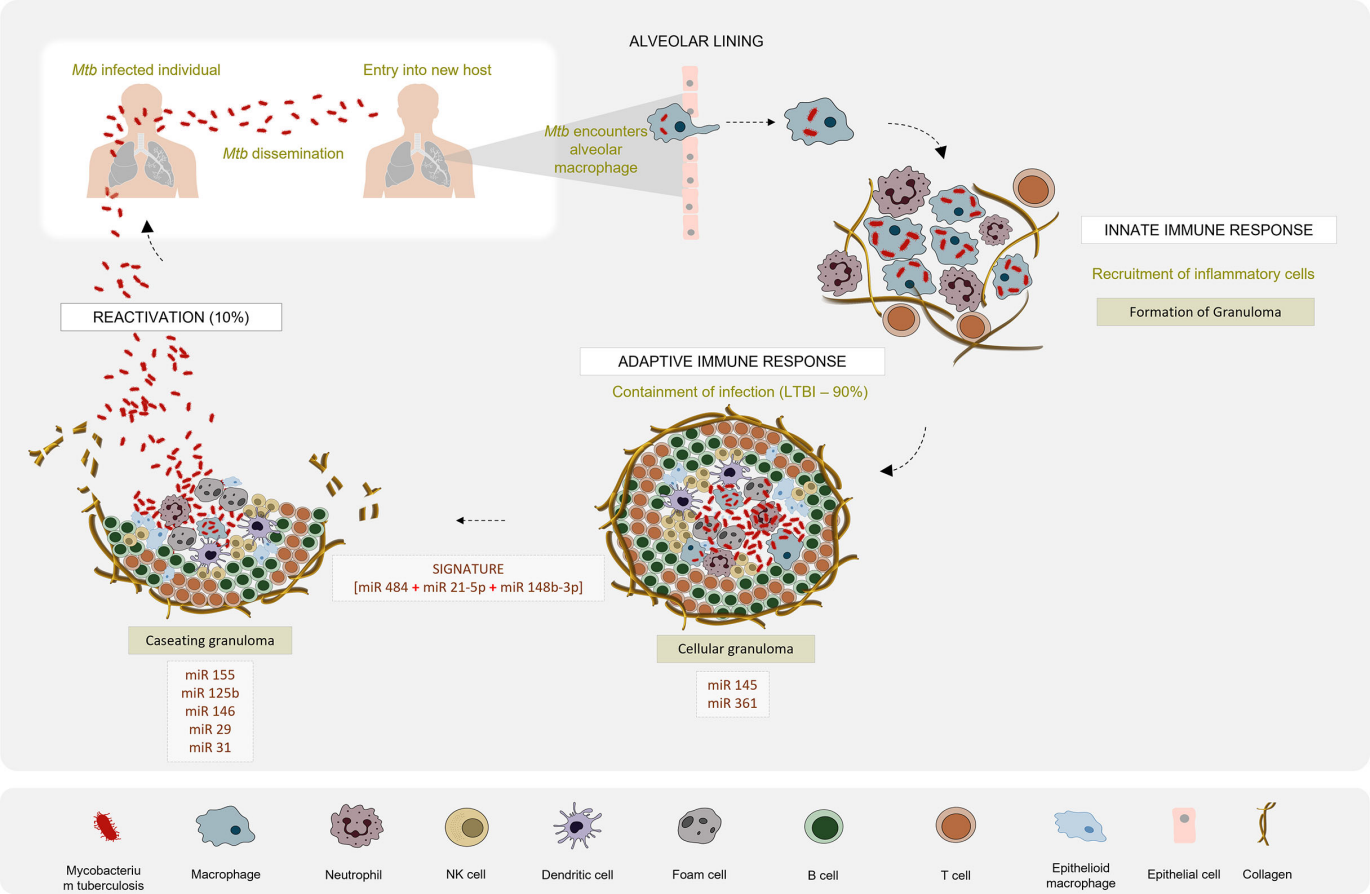
Differential miRNA expression profile in the pathogenesis of Tuberculosis: *Mtb* infection is initiated when tubercle bacilli from an active TB patient are disseminated through aerosol droplets to a new host. Resident alveolar macrophages in the alveolar space are the first to encounter and phagocytose the ingested *Mtb*. If *Mtb* manages to survive this first line of defence, it begins to gain access to the lung interstitial tissue. Dendritic cells also uptake *Mtb* and transport it to the thoracic lymph nodes for priming a T cell response. These events induce the recruitment of T and B cells and neutrophils, to the site of infection. They aggregate to form a granuloma with the infected macrophage in the centre surrounded by a lymphocytic cuff. Inside the granuloma, the production of a repertoire of pro- and anti-inflammatory cytokines aids the survival of *Mtb*. Successful evasion of the host immune response leads to a calcified granuloma, and establishment of latency. Active TB disease would ensue if resuscitation occurs and the granuloma starts caseating. Differential expression patterns of miRNAs reported in various studies between the latent and active forms of TB are depicted.

Recent reports suggest that most of the cellular immune response elicited by the host and the immune modulation strategies executed by *Mtb* are regulated by miRNAs (31). Several studies have reported dysregulated expression profiles of miRNAs that are intricately engaged in the host-pathogen interaction in active TB patients as compared to latently infected individuals and healthy controls (**Figure 2**). In this article, we reviewed the existing literature on the potential of miRNAs to serve as diagnostic biomarkers for TB and evaluated the diagnostic accuracy of reported miRNAs so as to identify a candidate miRNA biomarker that can be used as a reliable TB diagnostic marker. This systematic review has been registered in PROSPERO (CRD42021226559).

## Methods

### Search strategy

We have performed and reported this systematic review and meta-analysis in compliance with the Preferred Reporting Items for Systematic Reviews and Meta-Analyses (PRISMA) guidelines (32). An extensive literature search was executed in PubMed and Ovid interface to retrieve articles from Medline and Embase databases. Cochrane library was also included in the search platform. MeSH terms and key words related to tuberculosis and miRNA were applied for the search and are described in **Supplementary Tables 1A–C**. We also performed forward and backward reference checking from relevant review articles to identify any additional eligible studies.

### Eligibility criteria

#### Time Period

Articles published in English from January 2000 till December 2020 were included.

#### Study types

All study designs (cross-sectional, case-control or cohort studies) were considered regardless of prospective or retrospective sampling. Non-original articles including narratives and systematic reviews, meta-analyses, and conference abstracts were not included for consideration. Animal studies were also excluded.

#### Biomarker criteria

Studies reporting individual miRNAs or miRNA panels, or both were included. Studies evaluating other small RNAs were not considered. Articles evaluating miRNA as biomarker for other respiratory diseases and diseases other than tuberculosis were excluded as they are beyond the scope of this systematic review. Studies lacking sensitivity, specificity, and area under the curve (AUC) data were not included.

#### Study population

There were no exclusion criteria for patient characteristics, both adult and paediatric population studies were included.

### Screening and data extraction

All study articles retrieved by the search were fetched in Endnote X9 and duplicates were removed. We used Rayyan to manage the selection of studies (33). Publications from the electronic searches were initially subjected to screening of title and abstracts by two reviewers (ED, BS) to identify potentially eligible studies. Following this, the two authors independently performed full text screening to filter relevant studies. For certain articles with insufficient or incomplete information to enable a decision regarding inclusion/exclusion, the authors of the relevant papers were contacted for missing data. We collected details on the publication year, country, sample size (cases and controls separately), male/female proportion, mean or median age, study population (Active TB/LTBI/Healthy), reference test, sample source, screening and validation methods used, miRNA and/or miRNA signatures identified and diagnostic accuracy measures (sensitivity, specificity, AUC). We designed a data table and entered the extracted data into an excel database. We documented the values of True Positives (TP), False Positives (FP), True Negatives (TN), and False Negatives (FN) of the index test results. If not explicitly reported, the values were calculated from the reported sensitivity, specificity, and the sample size. For studies lacking sufficient data to construct a 2 x 2 table, the study authors were contacted requesting the required details. If study authors were unable to provide the information, we did not include the study for meta‐analysis but retained it in the narrative section for qualitative analysis alone. The extracted data were verified by two reviewers (HV and SV), with discrepancies discussed until consensus was reached.

### Quality assessment of the included studies

#### Evaluation using QUADAS-2 tool

All the included studies were assessed for their methodological quality using the Quality Assessment of Diagnostic Accuracy Studies (QUADAS-2) tool. The QUADAS-2 tool encompasses four domains - patient selection, index test, reference standard, and flow and timing to assess the risk of bias and clinical applicability of the studies. Each domain consists of signalling questions for which the bias and applicability was judged as “low”, “high”, or “unclear”. If the study provided vital information including the method used for identifying the biomarkers and the proper classification of the study cohort (Active TB or LTBI and control group), then the quality of the study was assessed as good. The seven items in QUADAS-2 were independently answered by three authors (ED, BS, and HV), and discrepancies were resolved by SV.

#### Adherence to STARD 2015 criteria

The STARD (Standards for Reporting of Diagnostic Accuracy studies) guidelines were framed to improve the transparency, quality, and completeness in reporting diagnostic studies (34, 35). The 30-item checklist consisting of sub-criteria were examined by reviewers BS and SV in tandem and 5 items were removed from the analysis due to lack of relevance in evaluating miRNAs as diagnostic biomarkers. Each criterion in the checklist was answered with “yes”, “no”, or “not applicable” by the two reviewers independently, and then compiled through consensus to provide the final score for each study. The checklist is provided in the **Supplementary Data section**.

### Data synthesis and statistical analysis

All computations were performed using R software (36). KT performed all the statistical analysis. Owing to limited number of studies, a random effects model based on inverse variance approach was applied for the analysis. Funnel plots were generated to visualize biased studies based on Egger’s test. Forest plots were constructed to demonstrate variability of point estimates between studies, their 95% confidence intervals (CI), as well as the weight of the sample sizes. Heterogeneity between studies was evaluated using I square with a cut-off point of ≥ 50% and a p value <0·10 and the degree of heterogeneity was defined by τ square test.

## Results

### Characteristics of the included studies

**Figure 3:**
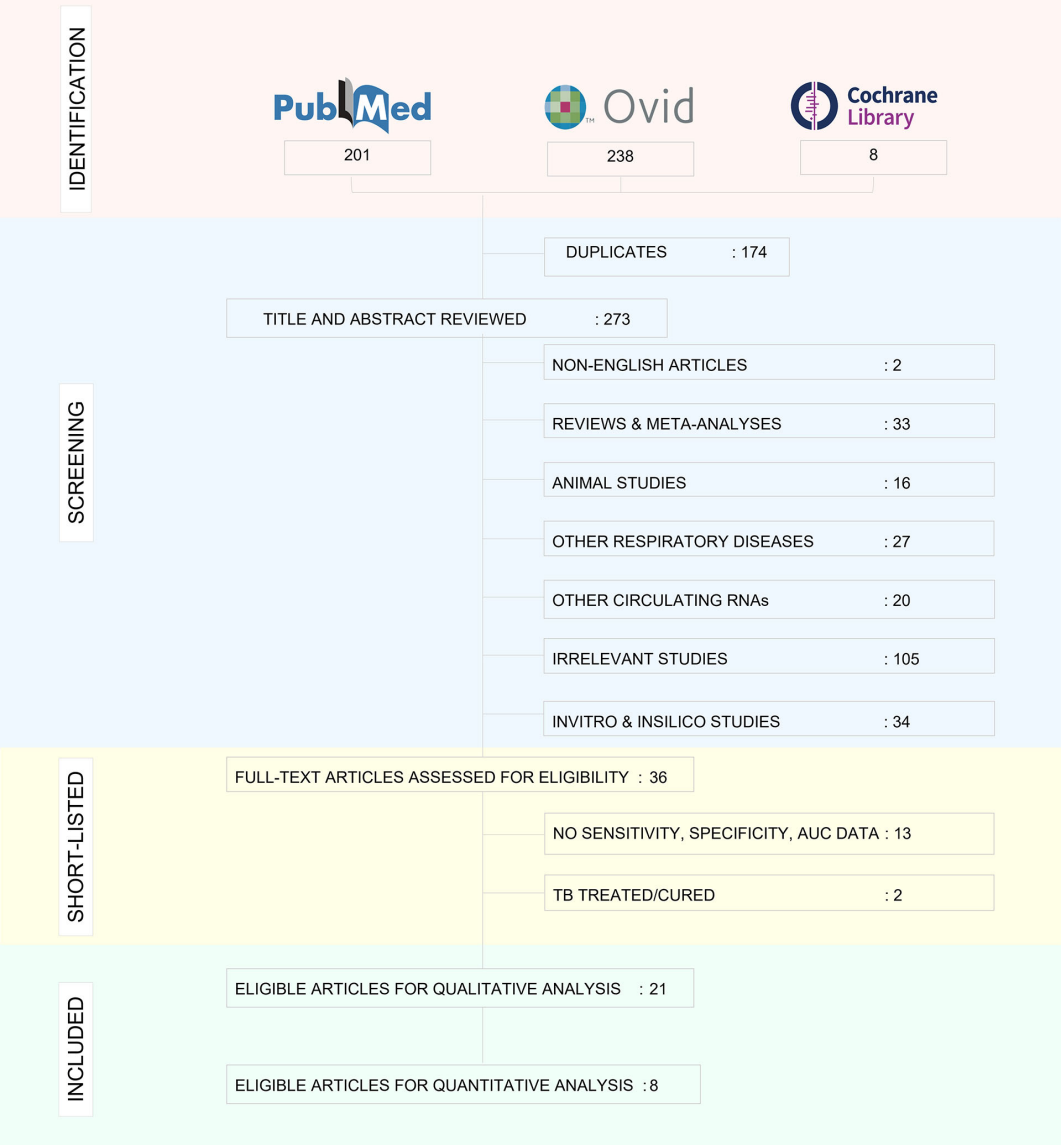
PRISMA flowchart showing study selection process.

The decision‐making process for including studies in the review is outlined in a PRISMA flowchart (**Figure 3**) and the study characteristics are tabulated in **Table 1**. 21 studies were included for qualitative analysis and five studies were excluded due to lack of sensitivity and specificity data. Included articles were published between 2011 and 2020 describing study samples collected between 2011 and 2018. 85·7% of studies were carried out in high TB incident countries (57·1% China; 14·3% Africa; 14·3% India) and 14·3% studies were from countries with low to moderate TB incidence (1 from Spain, 1 from Italy and 1 from Iran). Two studies were multi-centric. Majority of the studies focused on biomarkers for diagnosis of active TB and differentiation from healthy controls and/or latently infected individuals. Two studies specified miRNA biomarkers for LTBI diagnosis. One study provided a miRNA signature for predicting progression from LTBI to active TB.

**Table 1:** Characteristics of the included studies.

Authors	Year	Country	Patient characteristics						Diagnosis
			Control	Male/Female	Age*	Cases	Male/Female	Age*	
Abd et al. (37)	2013	Egypt	37 HC	21/16	50.1 ± 14.2	29 ATB	17/12	47.7 ± 9.8	TST
Alipoor et al (38)	2019	Iran	25 PTB	12/13	41 (15–65)	25 ATB	12/13	41 (15-65)	Clinical signs, Radiological findings, Sputum smear microscopy, Culture, NAAT
Chakrabarty et al (39)	2019	India	15 HC	8/7	45.33 (24-72)	15 ATB	11/4	47.67 (22-73)	Clinical signs, Radiological findings, Sputum smear microscopy, Culture, NAAT
Cui et al (40)	2017	China	64 HC	34/30	42.3 (17.41)	128 TB	85/43	43.3 (18.26)	
						28 DR-TB	17/11	44.3 (20.29)	
						33 DR-TB	23/10	44.7 (21.09)	
Duffy et al (41)	2018	South Africa, Uganda	54 HC			54 Progressors			Clinical signs, Culture, Radiological findings, sputum smear microscopy, TST
Fu et al (42)	2020	China	50 HC	29/21	33.2 ± 6.91	40 LTBI	22/18	31.95 ± 8.14	quantiFERON test
						60 ATB	38/22	32.07 ± 8.07	Clinical signs, Sputum smear microscopy, Culture
Fu et al (43)	2011	China	55 HC	35/20	39.05 ± 19.72	75 ATB	48/27	43.88 ± 21.04	Clinical signs, Radiological findings, Sputum smear microscopy, Culture
Latorre et al (44)	2015	Spain	16 HC	6/10	36.63 ± 8.42	17 LTBI	10/7	36.82 ± 8.77	TST, IGRA
						17 ATB	11/6	38.94 ± 15.44	Culture
Kathirvel et al (45)	2020	India	30 HC	21/9	10 (7-12)	30 ATB	18/12	8 (3.8-11)	Clinical signs, Radiological findings, Sputum smear microscopy, Culture, NAAT
Miotto et al (46)	2013	Italy, Uganda, Tanzania	28 HC			28 ATB			Sputum smear microscopy, Culture, NAAT
Ndzi et al (47)	2018	Cameroon	42 HC	13/29	27 ± 7	35 LTBI	8/27	34 ± 11	QuantiFERON test
			83 ATB			57/26	33 ± 12		Clinical signs, Sputum smear microscopy, Culture
Qi et al (48)	2012	China	65 HC	35/30	45.3 ± 20.9	30 ATB	18/12	44 ± 14.2	Clinical signs, Radiological findings, Sputum smear microscopy, Culture
Tu et al (49)	2019	China	41 HC	20/21	38.28 ± 10.25	60 ATB	24/36	37.5 ± 15.44	Clinical signs, Radiological findings, Sputum smear microscopy, Culture
Wagh et al (50)	2016	India	30 HC	18/12	28.71 ± 6.34	30 ATB	25/5	32.46 ± 12.03	Clinical signs, Radiological findings, Sputum smear microscopy, Culture
Wang et al (51)	2018	China	48 HC	26/22	32 ± 8.7	97 ATB	52/45	35 ± 6.3	Clinical signs, Radiological findings, Sputum smear microscopy, Culture, Biochip test
Wang et al (52)	2015	China	60 HC	35/25	2-11	65 ATB	38/27	1-10	
Wu et al (53)	2011	China	19 HC	11/8	37 (23-56)	21 ATB	16/5	49 (21-85)	Clinical signs, Radiological findings, Sputum smear microscopy, Culture, IGRA: QFT-GIT, T-SPOT.TB
Ying et al (54)	2020	China	32 Pneumonia	19/13	56.9 ± 29.4	68 ATB	39/29	46.8 ± 28.3	Clinical signs, Radiological findings, Sputum smear microscopy, Culture, ELISpot
			28 Lung cancer	18/10	61.5 ± 20.6				
			12 Unexplained pulmonary nodules	3/9	41.8 ± 17.6				
			50 HC	25/25	52.4 ± 14.7				
Zhang et al (55)	2019	China	20 HC			20 ATB			
Zhang et al (56)	2013	China	88 HC	65/23	48 (24-67)	108 ATB	73/35	45 (14-62)	Clinical signs, Radiological findings, Sputum smear microscopy, Culture, TST
			30 Lung cancer	23/7	57 (30-69)				
			30 Pneumonia	16/14	43 (23-64)				
			30 COPD	13/17	68 (57-80)				
Zhou et al (57)	2016	China	24 HC			28 ATB	16/12		Clinical signs, Radiological findings, Sputum smear microscopy, Culture, NAAT, TST, IGRA

HC, Healthy Control; ATB, Active TB; LTBI, Latent Tuberculosis; COPD, Chronic Obstructive Pulmonary Disease; TST, Tuberculin Skin Test; IGRA, Interferon Gamma Release Assay; QFT-GIT, QuantiFERON Gold-In-Tube; ELISpot, Enzyme-linked immune absorbent spot; NAAT, Nucleic Acid Amplification Test; *Age is given as either mean ± SD or mean (range) or mean (SD) as reported in each study.

### Characteristics of the study samples

The sample size ranged from 15 to 189 in the ATB cohort, 54 to 118 in the LTBI cohort and 15 to 178 in the healthy control cohort. Three studies comprised paediatric TB patients exclusively, 1 study had a combination of adult and paediatric populations, while the rest were adult patient studies. Ten studies used serum, three used plasma, three used PBMCs, two used whole blood, one used sputum, one used urine, and one used exosomes. miRNA screening was based on either literature or RNA sequencing or microarray, or miRNA databases. Validation of the significant miRNAs was carried out by quantitative real-time polymerase chain reaction (qRT-PCR) in all studies. We identified a total of 31 individual miRNAs and 12 miRNA signatures from 21 eligible studies (**Table 2**).

**Table 2:** Data extracted from included studies to perform qualitative and quantitative analysis.

Authors	Sample used	Screening method	Validation method	Biomarker utility	miRNA profile	AUC (95% CI)	Sensitivity (95% CI)	Specificity (95% CI)
Abd et al (37)	Serum	Literature	qRT-PCR	ATB vs HC	miR-197 ↑	0.96 (0.91-1.00)	1.00 (0.88-1.00)	0.95 (0.81-0.99)
Alipoor et al (38)	Serum exosomes	Literature	qRT-PCR	ATB vs HC	miR-484 ↑	0.73 (0.67-0.77)		
					miR-425 ↑	0.67 (0.58-0.75)		
					miR-96 ↑	0.63 (0.53-0.71)		
					miR-484, miR-425, miR-96	0.78 (0.73-0.83)		
Chakrabarty et al (39)	Serum	RNA-Seq (Ion Proton)	qRT-PCR	ATB vs HC	miR-125b-5p ↑	0.89 (0.66-0.99)	1.00 (0.78-1.0)	0.70 (0.44-0.92)
					miR-146a-5p ↑	0.79 (0.56-0.93)	1.00 (0.78-1.0)	0.56 (0.26-0.78)
					Combination	0.80 (0.67-0.89)	1.00 (0.78-1.0)	0.57 (0.32-0.83)
Cui et al (40)	Plasma	RNA-Seq (Illumina)	qRT-PCR	ATB vs HC	miR-769-5p ↓	0.92		
					miR-320a ↓	0.84		
					miR-22-3p ↓	0.71		
					Combination	0.91		
				Drug-Resistant TB vs Drug- Susceptible TB	miR-320a ↑	0.88		
Duffy et al (41)	Serum	miRNA PCR Panel (Exiqon)	qRT-PCR	LTBI to ATB Progression	miR-484, miR-21-5p, miR-148b-3p	0.67 (0.55-0.80)	0.59 (0.45-0.72)	0.73 (0.58-0.83)
Fu et al (42)	Serum	Literature	qRT-PCR	ATB vs HC	miR-145	0.93	0.95 (0.86-0.99)	0.86 (0.73-0.94)
				LTBI vs HC		0.82	0.83 (0.67-0.93)	0.76 (0.62-0.87)
				ATB vs LTBI		0.79	0.88 (0.77-0.95)	0.75 (0.59-0.87)
Fu et al (39)	Serum	Microarray (Exiqon)	qRT-PCR	ATB vs HC	miR-29a ↑	0.83	0.83 (0.72-0.90)	0.80 (0.67-0.90)
Latorre et al (44)	Whole Blood	Microarray (Agilent)	qRT-PCR	ATB vs LTBI+HC	miR-21, miR-194, miR-29c, miR-150	0.9 (0.89-0.90)	0.91 (0.64-0.99)	0.88 (0.62-0.98)
Kathirvel et al (45)	Plasma	Literature, Bioinformatics tools (micro T-CDS v5.0, miRTarBase v6.0, miRDB, TargetScanHuman v6.2)	qRT-PCR	ATB vs HC	miR-31↑	0.98 (0.95-1.00)	0.93 (0.78-0.99)	0.97 (0.83-0.99)
					miR-155 ↑	0.95 (0.89-1.00)	0.90 (0.73-0.98)	0.90 (0.73-0.98)
					miR-146a ↓	0.90 (0.82-0.98)	0.83 (0.65-0.94)	0.87 (0.69-0.96)
Miotto et al (46)	Serum	TaqMan Low Density Array	qRT-PCR	ATB vs HC European specific signature	let-7e, miR-148a, miR-16, miR-192, miR-193a-5p, miR-25, miR-365, miR-451, miR-590-5p, miR-885-5p	0.83 (0.68-0.92)	0.78 (0.55-0.91)	0.89 (0.67-0.97)
				ATB vs HC African specific signature	let-7e, miR-146a, miR-148a, miR-192, miR-193a-5p, miR-451, miR-532-5p, miR-590-5p, miR-660, miR-885-5p, miR223*, miR-30e	0.95 (0.76-0.99)	1.00 (0.72-1.00)	0.90 (0.60-0.98)
				ATB vs HC Pooled signature	let-7e, miR146a, miR-148a, miR-16, miR-192, miR-193a-5p, miR-25, miR365, miR-451, miR-532-5p, miR-590-5p, miR-660, miR-885-5p, miR-223*, miR-30e	0.82 (0.70-0.90)	0.86 (0.69-0.94)	0.79 (0.60-0.90)
Ndzi et al (47)	Plasma	Literature, miRNA disease databases: PhenomiR 2.0, HMDD 2.0, miR2disease	qRT-PCR	ATB vs HC	miR-29a-3p ↑	0.81	0.80 (0.69-0.88)	0.71 (0.55-0.84)
					miR-155-5p ↑	0.71	0.80 (0.69-0.88)	0.50 (0.34-0.66)
					miR-361-5p ↑	0.78	0.88 (0.79-0.94)	0.57 (0.41-0.72)
				LTBI vs HC	miR-29a-3p ↑	0.84	0.80 (0.69-0.88)	0.80 (0.63-0.92)
					miR-361-5p ↑	0.69	0.56 (0.44-0.66)	0.83 (0.66-0.93)
Qi et al (48)	Serum	TaqMan Low Density Array	qRT-PCR	ATB vs HC	miR-361-5p ↑	0.85		
					miR-889 ↑	0.77		
					miR-576-3p ↑	0.71		
					Combination	0.86		
Tu et al (49)	Serum	RNA-Seq (Illumina)	qRT-PCR	ATB vs HC	miR-17-5p ↑	0.82		
					miR-20b-5p ↑	0.75		
					miR-423-5p ↑	0.64		
					Combination	0.91	0.84 (0.71-0.92)	0.71 (0.54-0.84)
Wagh et al (50)	Serum	Literature	qRT-PCR	ATB vs HC	miR-16 ↑	1.00 (1.00-1.00)		
					miR-155 ↑	0.97 (0.92-1.04)		
					miR-29a ↑	0.68 (0.54-0.82)		
					miR-125b ↑	0.51 (0.36-0.66)		
Wang et al (51)	Urine	Cytoscape v3.2.1, Target prediction algorithms: Targetscan, Miranda, MicroCosm, PicTar	qRT-PCR	ATB vs HC	miR-625-3p ↑	0.86	0.84 (0.75-0.91)	0.83 (0.61-0.95)
					miR-155 ↑	0.67	0.60 (0.44-0.75)	0.57 (0.34-0.78)
Wang et al (52)	PBMC	Literature	qRT-PCR	ATB vs HC	miR-31 ↓	0.97 (0.93-0.99)	0.99 (0.92-0.99)	0.87 (0.75-0.94)
Wu et al (53)	PBMC	Microarray (Agilent)	qRT-PCR	ATB vs HC	miR-155 ↑	0.90 (0.80-0.99)	0.48 (0.26-0.7)	0.95 (0.74-0.99)
					miR-155* ↑	0.79 (0.66-0.93)	0.43 (0.22-0.66)	0.95 (0.74-0.99)
Ying et al (54)	Sputum	Microarray (Agilent)	qRT-PCR	ATB vs HC	miR-155 ↑		0.94 (0.86-0.96)	0.88 (0.76-0.94)
Zhang et al (55)	PBMC	GEO database	qRT-PCR	ATB vs HC	miR-892b ↓	0.77	0.55 (0.32-0.77)	0.90 (0.68-0.99)
					miR-199b-5p ↑	0.71	0.50 (0.27-0.73)	0.80 (0.56-0.94)
					miR-582–5p ↑	0.7	0.40 (0.19-0.64)	0.95 (0.75-0.99)
Zhang et al (56)	Serum	Solexa Sequencing (Illumina)	qRT-PCR	ATB vs HC/Pneumonia/Lung Cancer/COPD	miR-378 ↑	0.88 (0.82-0.93)	0.96 (0.91-0.99)	0.64 (0.53-0.73)
					miR-483-5p ↑	0.70 (0.63-0.77)	0.74 (0.67-0.82)	0.61 (0.50-0.71)
					miR-22 ↑	0.71 (0.64-0.78)	0.68 (0.58-0.75)	0.71 (0.59-0.79)
					miR-29c ↑	0.85 (0.79-0.90)	0.73 (0.64-0.8)	0.83 (0.73-0.89)
					miR-101 ↑	0.86 (0.80-0.91)	0.77 (0.68-0.84)	0.77 (0.67-0.85)
					miR-320b ↑	0.79 (0.72-0.86)	0.74 (0.66-0.82)	0.67 (0.56-0.76)
					Combination	0.98 (0.93-0.99)	0.95 (0.9-0.98)	0.92 (0.85-0.97)
Zhou et al (57)	Whole Blood	Microarray (Agilent)	qRT-PCR	ATB vs HC	miR-1 ↑	0.93 (0.81-0.98)	0.76 (0.55-0.89)	1.00 (0.86-1.0)
					miR-10a ↑	0.95 (0.84-0.99)	0.76 (0.55-0.89)	1.00 (0.86-1.0)
					miR-125b ↑	0.96 (0.86-0.99)	1.00 (0.88-1.0)	0.81 (0.58-0.93)
					miR-146a ↑	0.96 (0.86-0.99)	1.00 (0.88-1.0)	0.81 (0.58-0.93)
					miR-150 ↑	0.99 (0.90-1.00)	1.00 (0.88-1.0)	0.91 (0.73-0.99)
					miR-155 ↑	0.92 (0.80-0.98)	0.96 (0.82-0.99)	0.86 (0.68-0.97)
					miR-31 ↑	0.95 (0.84-0.99)	0.96 (0.82-0.99)	0.91 (0.73-0.99)
					miR-29b ↑	0.70 (0.54-0.98)	0.56 (0.37-0.76)	0.91 (0.73-0.99)
					Combination	0.99 (0.91-1.00)	0.96 (0.82-0.99)	1.00 (0.86-1.0)

AUC, Area under the curve; CI, Confidence Interval; qRT-PCR, quantitative Real Time PCR; ATB, Active TB; HC, Healthy Control; COPD, Chronic Obstructive Pulmonary Disease; LTBI, Latent Tuberculosis Infection; ↑ miRNA upregulated; ↓ miRNA downregulated.

### Qualitative analysis of the studies

#### QUADAS-2 and STARD Assessment

**Figure 4:**
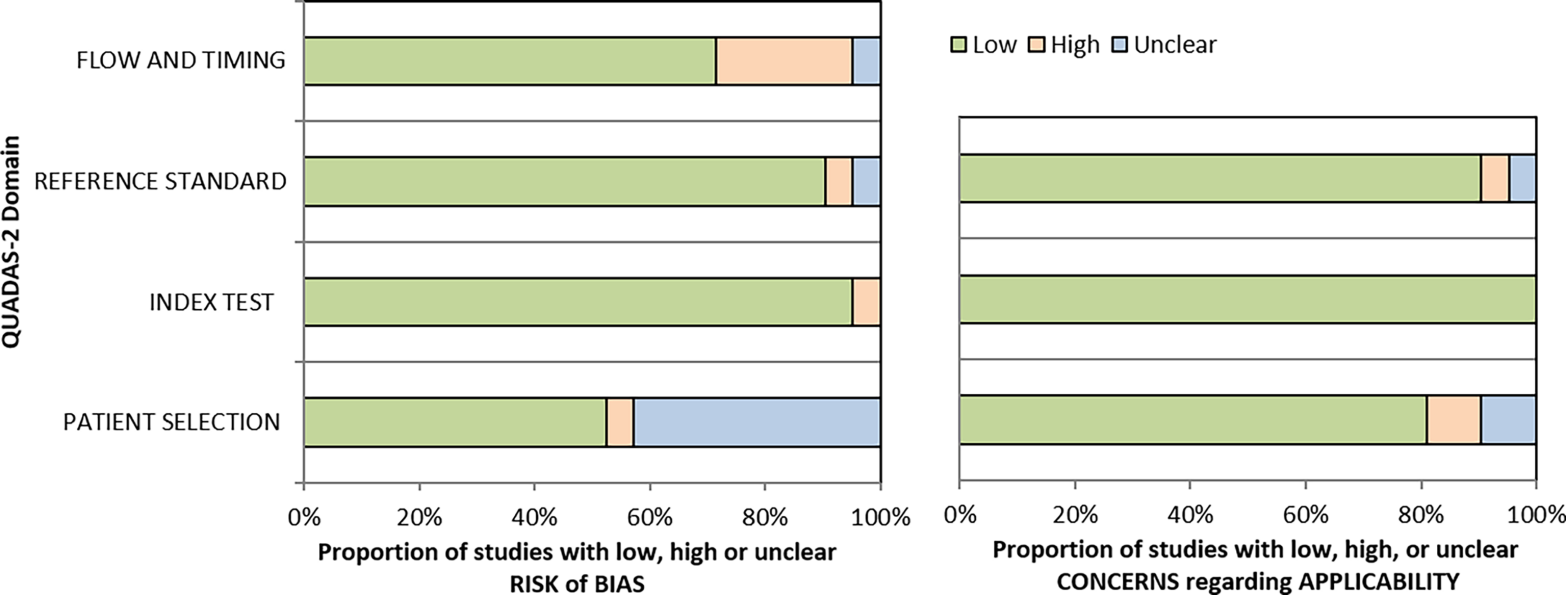
Summary of the results of QUADAS-2 assessment of selected studies.

The patient flow and selection led to considerable bias, but the overall risk of bias of the included studies assessed by QUADAS was low (<50%) **Figure 4**. The applicability concerns were very low in all four domains (All 21 included studies exhibited adherence to the STARD guidelines with a moderate score (**Supplementary Data**).

### Quantitative analysis of the studies

The first statistical approach in meta-analysis is to assess the consistency of the reported miRNAs for diagnosis of active TB and LTBI by analysing the heterogeneity. A funnel graph was plotted to assess the likelihood of study bias. An asymmetric distribution of data points was observed with p>0·05 indicating the existence of potential publication bias (**Supplementary Figure 1**). Forest plots also indicated high heterogeneity (I^2^>50%). This bias is mainly attributed to inconsistency in the reported miRNAs and small sample size, therefore we selected miRNAs that were reported in at least two studies to ensure reliability of the miRNA as a candidate biomarker. Hence, we included only eight studies for the meta-analysis.

### Accuracy of miRNAs as biomarkers of TB diagnosis

**Figure 5:**
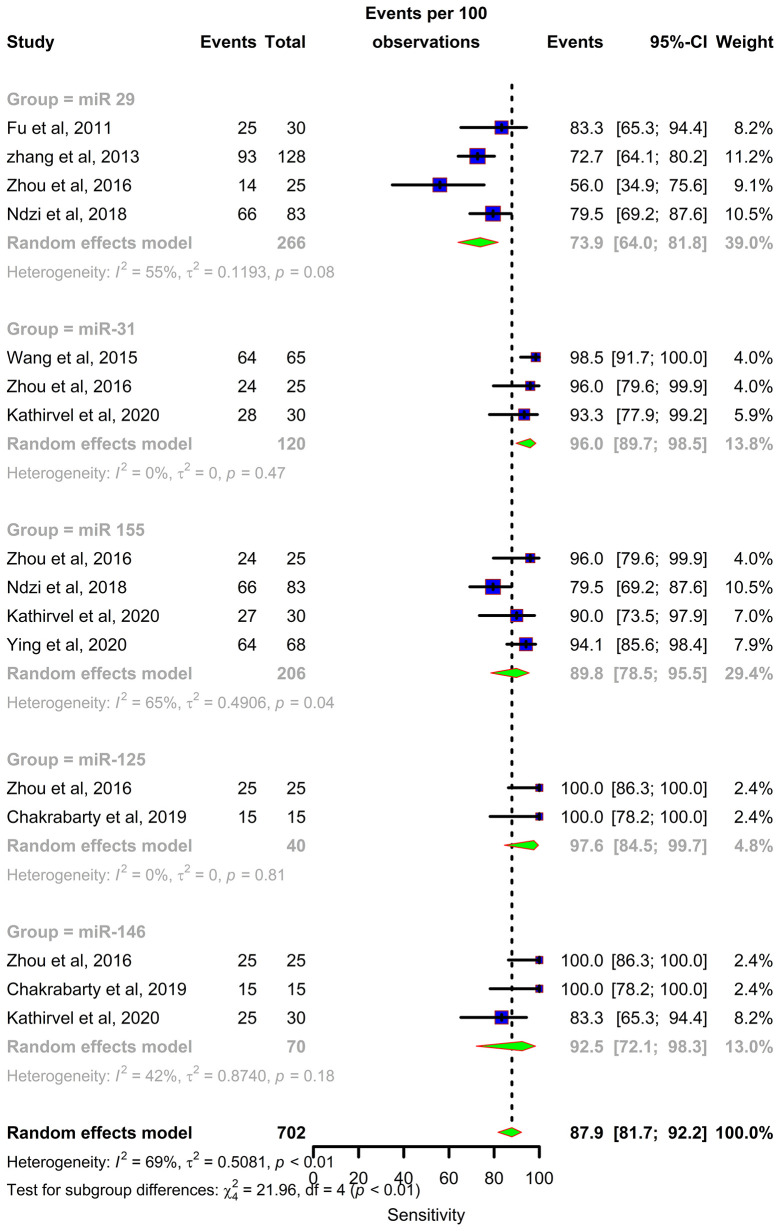
Sensitivity forest plot: Forest plot depicting sensitivity of miR 29 family (miR-29a, 29b, 29c), miR 31, miR 125b, miR 146a and miR 155.

**Figure 6:**
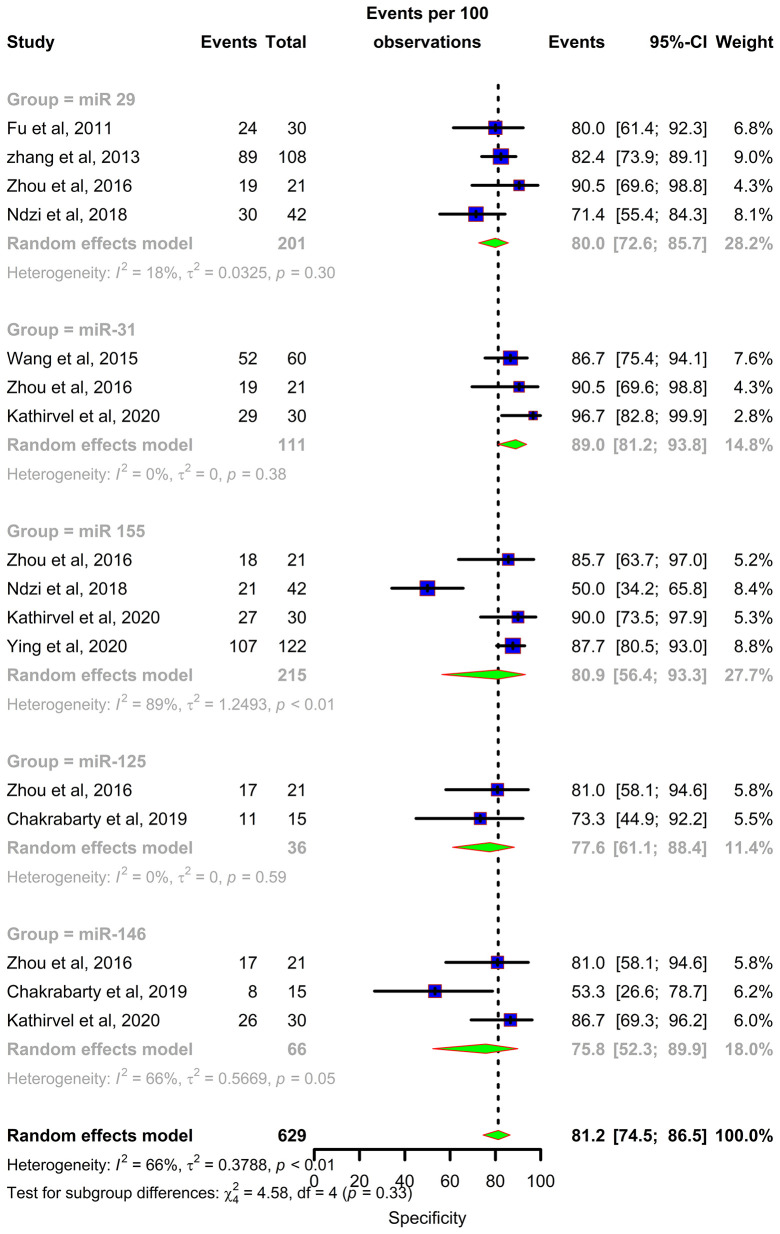
Specificity forest plot: Forest plot depicting specificity of miR 29 family (miR-29a, 29b, 29c), miR 31, miR 125b, miR 146a and miR 155.

**Figure 7:**
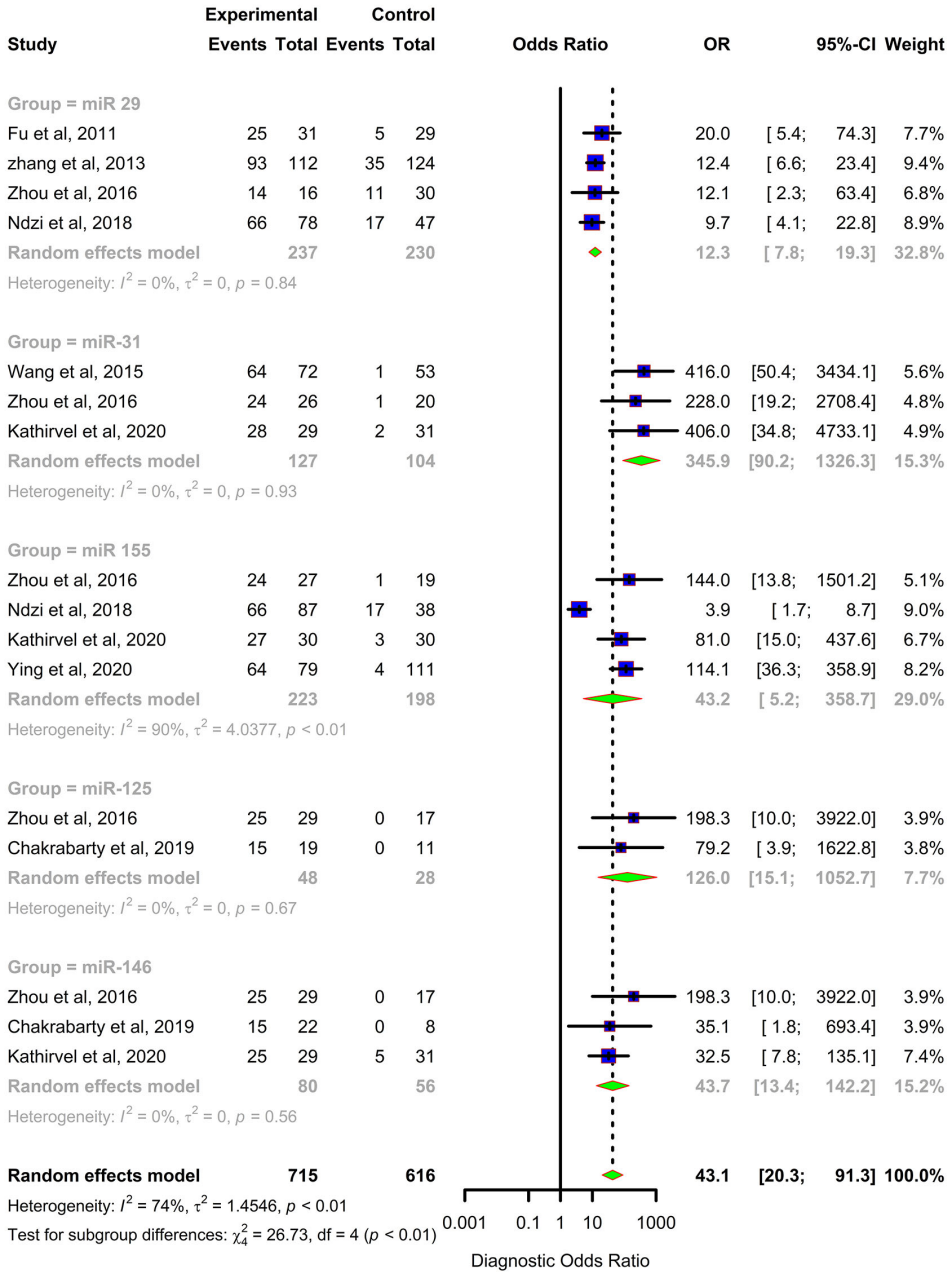
Diagnostic Odds Ratio (DOR) forest plot: Forest plot depicting Diagnostic Odds Ratio (DOR) of miR 29 family (miR-29a, 29b, 29c), miR 31, miR 125b, miR 146a and miR 155.

Forest plots were constructed for each miRNA group to assess the heterogeneity of the studies and determine the overall pooled values of sensitivity, specificity, and diagnostic odds ratio (DOR). Forest plots for miR-29 family (29a, 29b & 29c) revealed a sensitivity of 73·9% (95% CI 64-81·8), specificity of 80% (95% CI 72·6-85·7), and DOR of 12·3 (95% CI 7·8-19·3). The heterogeneity was less than 50% except for the sensitivity (I^2 =^ 55%). miR-31 exhibited a sensitivity of 96% (95% CI 89·7-98·5), specificity of 89% (95% CI 81·2-93·8), and DOR of 345·9 (95% CI 90·2-1326·3). A sensitivity of 97·6% (95% CI 84·5-99·7), specificity of 77·6% (95% CI 61·1-88·4), and DOR of 126 (95% CI 15·1-1052·7) was shown by miR-125b. Both mir-31 and miR-125b did not show any heterogeneity (I^2 =^ 0%) in the sub-group analysis. miR-146a had a sensitivity of 92·5% (95% CI 72·1-98·3), specificity of 75·8% (95% CI 52·3-89·9), and DOR of 43·7 (95% CI 13·4-142·2) with a heterogeneity (I^2^) of 66% for specificity. The sensitivity, specificity, and DOR of miR-155 was 89·8% (95% CI 78·5-98·5), 80·9% (95% CI 56·4-93·3), and 43·2 (95% CI 5·2-358·7) respectively with high heterogeneity. The subsequent step was to combine the effect size of each study. The overall pooled sensitivity and specificity of the above miRNAs were 87·9% (95% CI 81·7-92·2) (**Figure 5**) and 81·2% (95% CI 74·5-86·5) (**Figure 6**), while the DOR was 43·1 (95%CI 20·3-91·3) (**Figure 7**).

## Discussion

A key component of the interim milestone for 2035 set by WHO for the End TB strategy is early diagnosis of the disease through systematic screening of high-risk groups. This calls for the identification and validation of definite biomarkers that discriminate active and latent TB and also predict progression to disease. Circulating miRNAs are potent biomarkers for TB diagnosis. miRNAs have also been explored as biomarkers for distinguishing between latent and active tuberculosis (47, 58, 59), as well as TB with HIV coinfection (46, 60). Emerging studies also demonstrate their promising role in predicting progression (41). miRNAs elicit an integral role in framing the innate and adaptive immunity during *Mtb* infection, by influencing gene expression in macrophages, dendritic cell, B and T cells. Multiple *in vitro* and *in vivo* studies have demonstrated the role of miRNAs in host immune regulatory mechanisms during TB pathogenesis, including apoptotic pathway trigger, autophagy induction, IFN γ stimulation and TNF α secretion. *Mtb* employs various strategies to combat these regulatory mechanisms and builds up its survival.

**Figure 8:**
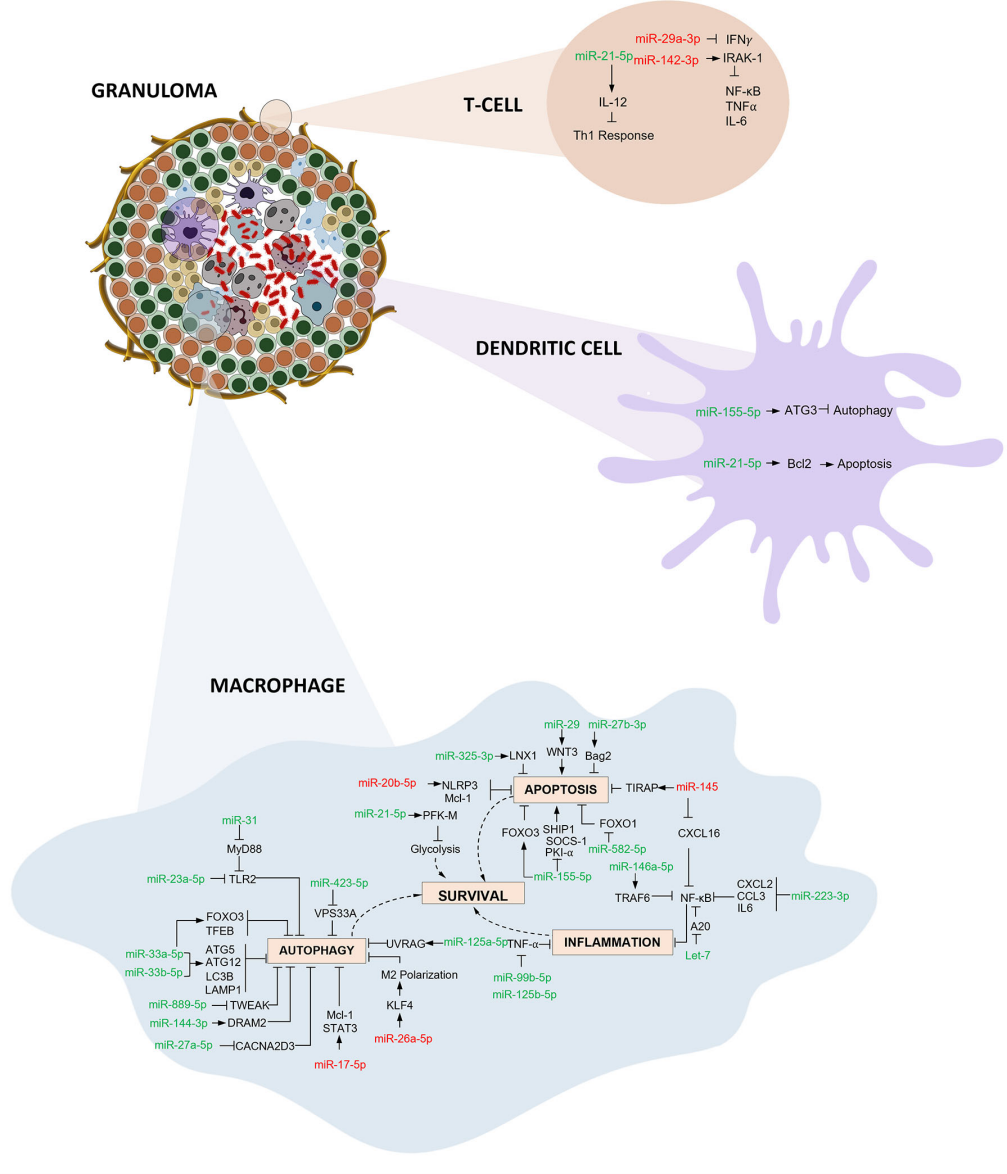
Immunoregulatory mechanisms involving miRNA in macrophages, dendritic cells and T cells: Three primary mechanisms of immune regulation are documented: (i) Autophagy evasion: Autophagy can degrade intracellular *Mtb* by autolysosomes. *Mtb* regulates the expression of various miRNAs thereby inhibiting autophagy activation and aiding its survival inside macrophages. (ii) Apoptosis impairment: Host macrophages combat infection by eliminating niche cells for *Mtb* growth and by packaging tubercle bacilli in apoptotic bodies. *Mtb* employs multiple strategies to circumvent this programmed cell death. (iii) Inflammation obstruction: *Mtb* infection initiates the activation of various components of the immune system resulting in recruitment of inflammatory cells. *Mtb* interferes with various downstream signalling events and inhibits the inflammatory responses thereby thriving within the hostile environment. (miRNAs shown in red are downregulated and green are upregulated).

In this systematic review and meta-analysis, we evaluated the diagnostic accuracy of miRNAs as biomarkers for Tuberculosis. The overall analysis demonstrates moderate diagnostic accuracy with a DOR of 43·1. We conducted meta-analysis for miR 29 family (miR-29a, 29b, 29c), miR 31, miR 125b, miR 146a, and miR 155. Among these, miR 31 is specific for diagnosis of paediatric TB with a sensitivity of 96% (95% CI 89·7-98·5) and specificity of 89% (95% CI 81·2-91·2). Elevated levels of miR-31 suppresses MyD88, a TLR-2 adaptor protein and PP2A, thus negatively regulating the host’s innate immunity against *Mtb* (61). miR-31 also inhibits production of pro-inflammatory cytokines like IL-6, TNF-α, and IFN-γ which facilitate the induction of MTOR-responsive WNT and SHH pathways, resulting in inhibition of IFN γ-mediated autophagy (62). On the contrary, Wang et.al. reported decreased levels of miR-31 expression and significantly higher levels of serum IL-6, TNF-α, NF-κB, and IFN-γ in children with TB (52). However, *in vitro* studies support the autophagy inhibition role through the upregulation of miR-31. miR-29 was found to be downregulated in IFN-γ producing cells like NK cells and CD4+ and CD8+ T cells after *Mycobacterium bovis* BCG infection in mice (63). In latent and active TB, miR-29 expression was significantly increased, leading to reduced IFN-γ levels and subsequent signal pathway modifications (64). miR-125b contributes to a suppressed inflammatory response by directly interacting with TRAF6, and subsequently mediates *Mtb* survival. Additionally, RAF1 inhibition by miR-125b leads to reduced levels of TNF-α, IL-6, etc., indicating miR- mediated immune escape of *Mtb* (65). miR-146 has a sensitivity of 92·5% (95% CI 72·1-98·3) but a constrained specificity of 75·8% (95% CI 52·3-89·9). Increased levels of miR-146a targets TRAF6 which modulates the suppression of NF-κB and MAPKs pathways thereby hindering iNOS expression and NO synthesis leading to mycobacterial survival (66). On the other hand, it can also lead to TNF-α attenuation and downregulation of potential targets like IRAK1, TRAF6, and PTGS2 (67). Thus, miR-146a has both positive and negative effects on *Mtb* survival. miR-155 harnesses macrophage and T-cell survival by SHIP1 mediated regulation. This coupled role provides a pro-bacterial environment during the early phase of infection. Increased survival of *Mtb*-specific T-cells contributes to increased protection against the bacteria (68). *Mycobacterium bovis* BCG infection is known to upregulate miR-155 expression through TLR2 and other signalling pathways. miR-155 dependent increase in ROS occurs due to the suppression of SHIP1 in BCG-infected macrophages (69). An exhaustive network profile of various miRNAs involved in the pathogenesis and survival of *Mtb* is depicted in **Figure 8**.

Despite these promising results, we did observe significant heterogeneity because of discrepancies among the different studies. Various factors starting from the type of sample, its storage and processing, miRNA profiling and validation methods, to data normalization could introduce potential bias and lead to inconsistency in results. Since the number of studies available for analysis was very less, we could not carry out any other subgroup analysis based on sample source or profiling methods. Some studies reported the -3p or -5p variant while some did not, hence we merged the variants. This could have decreased the accuracy of our analysis. Another major limitation is the small sample size in each study. For any biomarker discovery study, the size of the cohort is very crucial and should be representative of the target population. Though reported miRNA signatures had better accuracy than the individual miRNAs, we could not include them for our analysis since no two signatures were the same. Regardless of these limitations, ours is the first meta-analysis that comprehensively aggregated available data on miRNAs and analysed their utility in the field of tuberculosis.

Scientific advancements have paved the way for development of various novel miRNA detection platforms including Bead-Array based profiling, microfluidics, miRNA activity reporter assays and amplification assays [(loop-mediated isothermal amplification (LAMP), exponential isothermal amplification assay (EXPAR), rolling circle amplification (RCA) and strand displacement amplification (SDA)] (70) that can be exploited for the development of sensitive diagnostic tests for TB using the most promising candidate miRNAs. Using miRNAs as diagnostic reagents would help in the development of rapid and cost- effective tests for various infectious diseases, circumventing the disadvantages of conventional diagnostic assays. Yet, detecting weakly expressed miRNAs can be challenging, and this can be overcome by using highly sensitive detection platforms such as small RNA-seq method (71). In this line, higher background signals in fluorescent detection platforms can also pose a challenge and reduce the sensitivity of the test. Nanotechnology-based approaches such as the use of electrochemical biosensors are currently being explored to overcome these challenges (72).

The following observations were made during this comprehensive systematic review and meta-analysis: (i) A substantial between-platform discordance is inevitable since the miRNA spectrum between various sample sources are different, (ii) miRNA signatures have better diagnostic performance than single miRNAs, (iii) miRNA expression profile is age-specific; miR 31 specifically finds application in diagnosis of Paediatric TB (iv) Distinct miRNA profiles for different stages of TB is emerging. This can lead to the identification of a promising biomarker panel to predict progression from Latent Tuberculosis infection to Active TB disease.

## Conclusion

Early diagnosis of tuberculosis is crucial for achieving the WHO global End TB milestone. miRNAs are known to have potential diagnostic abilities for various diseases with demonstrated accuracy. Here we present a comprehensive overview of the immunological role of miRNAs in tuberculosis by systematically reviewing the available literature. We also performed a meta-analysis of five miRNAs and demonstrate their promising role in TB diagnosis. The overall diagnostic odds ratio (DOR) of the five miRNAs was 43·1. miR-31 individually exhibited the maximum DOR of 345·9 portraying very high diagnostic accuracy.

## Data availability statement

The original contributions presented in the study are included in the article/**Supplementary Material**. Further inquiries can be directed to the corresponding author.

## Author contributions

ED, BS, SV and HV collected and interpreted data. KT performed statistical analysis. LH designed the study. All authors contributed to the article and approved the submitted version.

## Funding

ED thanks Department of Science and Technology (DST), India for providing INSPIRE fellowship.

## Acknowledgments

The authors thank Mr. Bennett Henzeler, National Institute for Research in Tuberculosis for his help in designing the figures. 

## Conflict of interest

The authors declare that the research was conducted in the absence of any commercial or financial relationships that could be construed as a potential conflict of interest.

## Publisher’s note

All claims expressed in this article are solely those of the authors and do not necessarily represent those of their affiliated organizations, or those of the publisher, the editors and the reviewers. Any product that may be evaluated in this article, or claim that may be made by its manufacturer, is not guaranteed or endorsed by the publisher.
