# Supplementary material for: MicroRNAs as diagnostic biomarkers for Tuberculosis: A systematic review and meta- analysis
Source: Front Immunol. 2022 Sep 27;13:954396. doi: 10.3389/fimmu.2022.954396 (PMC9551313; doi:10.3389/fimmu.2022.954396)
Supplement: Supplementary file 1 [file DataSheet_1.pdf]

SUPPLEMENTARY DATA

Table of contents

- Supplementary table 1a: Search strategy used to retrieve published literature from Pubmed
- Supplementary table 1b: Search strategy used to retrieve published literature from Ovid
- Supplementary table 1c: Search strategy used to retrieve published literature from Cochrane
- STARD guidelines
- Supplementary figure 1: Funnel plot asymmetry test to assess publication bias

Supplemental table 1A: Search strategy used to retrieve published literature from PubMed

Search Item	Search term	Connecting operator
**Tuberculosis**	(Tuberculosis [MeSH] OR Mycobacterium tuberculosis [MeSH] OR (Tuberculosis OR TB OR Mycobacterium tuberculosis OR MTB OR "M. Tuberculosis"))	**AND**
**Diagnosis**	(Diagnosis [MeSH] OR Diagnosis [subheading] OR Prognosis [MeSH] OR (Diagnosis OR diagnostic OR detect* OR predict* OR prognosis OR prognostic OR screen* OR distinguish* OR differentiat* OR discriminat*))	**AND**
**Biomarkers**	miRNA* OR microRNA* OR miR OR "small RNA"	**AND**
**Performance**	(Area under Curve [MeSH] OR Sensitivity and Specificity [MeSH] OR (Area under curve OR area under curves OR AUC OR receiver operating characteristic OR ROC OR Accuracy OR Performance OR sensitivity OR specificity))	**AND**
**Time Period**	(2000:2020[pdat])	

Supplemental table 1B: Search strategy used to retrieve published literature from Ovid

Search Item	Search term	Connecting operator
**Tuberculosis**	Tuberculosis OR TB OR Mycobacterium tuberculosis OR MTB OR “M. Tuberculosis"	**AND**
**Diagnosis**	Diagnosis OR diagnostic OR detect* OR predict* OR prognosis OR prognostic OR screen* OR distinguish* OR differentiat* OR discriminat*	**AND**
**Biomarkers**	miRNA* OR microRNA* OR miR OR "small RNA"	**AND**
**Performance**	Area under curve OR area under curves OR AUC OR receiver operating characteristic OR ROC OR Accuracy OR Performance OR sensitivity OR specificity	**AND**
**Time Period**	2000:2020	

Supplemental table 1C: Search strategy used to retrieve published literature from Cochrane

Search Item	Search term
**#1**	MeSH descriptor: [Tuberculosis] explode all trees
**#2**	Tuberculosis OR Mycobacterium tuberculosis
**#3**	MeSH descriptor: [MicroRNAs] explode all trees
**#4**	MicroRNA or miRNA or Micro RNA or stRNA
**#5**	ROC Curve or diagnosis or diagnostic or specificity or sensitivity
**#6**	#1 OR #2
**#7**	#3 OR #4
**#8**	#5 AND #6 AND #7

STARD guidelines

STARD_Combined.xlsx

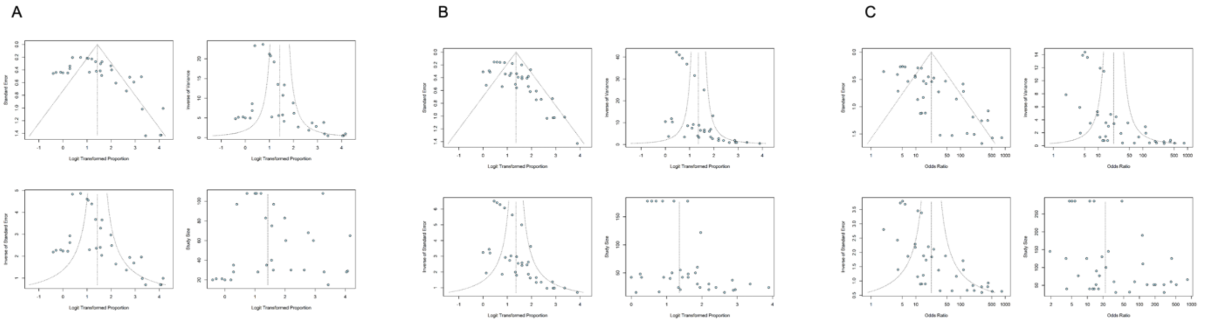

Supplemental Figure 1: Funnel plot asymmetry test to assess publication bias: Funnel plot is plotted for **(A)** Sensitivity **(B)** Specificity **(C)** Diagnostic Odds Ratio (DOR) of the 21 studies to show asymmetry of logit transformed proportion against standard error, inverse of variance, inverse of standard error and study size.
